# HIV-1 Expressing the Envelopes of Transmitted/Founder or Control/Reference Viruses Have Similar Infection Patterns of CD4 T-Cells in Human Cervical Tissue *Ex Vivo*

**DOI:** 10.1371/journal.pone.0050839

**Published:** 2012-12-06

**Authors:** Melanie Merbah, Anush Arakelyan, Tara Edmonds, Christina Ochsenbauer, John C. Kappes, Robin J. Shattock, Jean-Charles Grivel, Leonid B. Margolis

**Affiliations:** 1 Section of Intercellular Interactions, Program in Physical Biology, Eunice-Kennedy Shriver National Institute of Child Health and Human Development, National Institutes of Health, Bethesda, Maryland, United States of America; 2 University of Alabama at Birmingham, Birmingham, Alabama, United States of America; 3 Medicine, Imperial College, London, United Kingdom; University of Central Florida College of Medicine, United States of America

## Abstract

Recently, it was found that 80% of sexual HIV-1 transmissions are established by a single virion/viral genome. To investigate whether the transmitted/founder (T/F) viruses have specific biological properties favoring sexual transmission, we inoculated human cervical tissue explants with isogenic HIV-1 viruses encoding Env sequences from T/F and control reference (C/R) HIV-1 variants as well as with full length T/F HIV-1 and compared their replication efficiencies, T cell depletion, and the activation status of infected cells. We found that all the HIV-1 variants were capable of transmitting infection to cervical tissue *ex vivo* and in this system preferentially replicate in activated CD4 T cells and deplete these cells. There was no difference in the biological properties of T/F and C/R HIV-1 variants as evaluated in *ex vivo* cervical tissue.

## Introduction

The main HIV-1 gateway in male-to-female transmission is believed to be the cervico-vaginal mucosa where the infection of the first target cell(s) occurs, followed by a local viral amplification, which precedes the establishment of the systemic infection [1]. Recent data indicate that out of diverse HIV-1 quasispecies in the infecting partner, in more than 80% of clade B transmission cases, a single viral variant, predominantly of R5 phenotype, establishes infection [2], [3].

At least two hypotheses may explain this result: Either the transmission of a given variant is a stochastic process accompanied by mechanisms that prevent the transmission/amplification of other viruses, or transmitted viruses have specific traits to overcome the multiple “gatekeepers” of the vaginal mucosa. The recent isolation and cloning of T/F virus envelopes and full-length infectious clones enables the study of their properties under controlled conditions.

We used recently described [4] isogenic, replication-competent proviral constructs in which the *env* sequences encoding the ectodomain (gp120 and ectodomain of gp41) of the Env glycoporotein were derived from either T/F HIV-1 variants or chronic/reference (C/R) HIV-1 strains utilized as control viruses. We studied transmission of these viruses in a recently developed system [5] of collagen raft-supported blocks of human cervical tissue. To investigate the abilities of several HIV-1 strains to initiate infection in human cervical tissue *ex vivo*, we investigated the efficiencies of viral replication, the cellular targets of these viruses, and the target cell activation status.

## Materials and Methods

### HIV-1 Virus Strains

We recently described a molecular approach to express *env* sequences of interest *in cis* in isogenic, replication-competent, NL4-3-based backbones infectious molecular clones (Env-IMC), refered here as “NL-Env.ecto”. To avoid creating chimerism in any reading frames overlapping *env*, heterologous *env* sequences encoding the ectodomain were cloned into the NL4-3 backbone, wherein the recombinant viruses express full-length Env [4]. We used three R5-tropic reference HIV-1 variant (NL-SF162.ecto, NL-YU-2.ecto, and NL-BaL.ecto), as well as eight viruses encoding CCR5-utilizing *env* gene*s* from clade B mucosally transmitted T/F HIV-1 variants, including NL-CH077.ecto, NL-1051.TD12.ecto, NL-1051.C22.ecto, NL-TT31P.2G1.ecto, NL-TT31P.2F10.ecto, NL-SC22.3C2.ecto, NL-RHPA.ecto, and NL-9010.A1.ecto, with *env* genes derived, respectively, from subjects 700010077 (male; single variant transmission); 1051-12 (female; infected with two related variants); TT31P (female; infected with two related variants); SC22 (female; single variant transmission), RHPA4259 (female; single variant transmission); and 9010 (female; single variant transmission) as described by [2]). In parallel, we employed the full-length IMC, CH077.t and RHPA.c, which represent the T/F viruses from subjects 700010077 and RHPA4259, respectively [6]. We have previously established that the cellular tropism of Env-IMC closely match that of their respective matched full-length IMC or isolates [6]. The R5-tropic HIV-1_BaL_ (NIH AIDS Research & Reference Reagent Program, catalogue #510), isolated from a chronically infected human infant lung, served as another control virus.

Virus stocks were prepared essentially as described [4], [6]. Briefly, 293T cells were transfected with proviral DNA, medium was changed at 16 hours, and virus stocks harvested at 60 hours. HIV-1 BaL was grown in PBMC. All stocks were titered on TZM-bl cells, and infectious units (IU) per ml were determined by beta-galactosidase staining for quality assurance. Viral stocks were directly used for inoculation of tissues. TCID_50_ on TZM-bl cells of the different viruses varied from 1x10^7^ to 4.5x10^7^ (for the C/R HIV-1 variants the range was from 2.5 to 4.0 x10^7^ and for T/F HIV-1 variants it was from 1.0 to 4.5x10^7^). Such differences in TCID_50_ values measured in one system do not directly translate into consistent differences in virus replication capacity in another system, in this case in tissues from various donors [7]. Furthermore, the observed differences in TCID_50_ of different viruses are much less than the variability that is seen for replication of a given virus stock in tissues from different donors [5], [8].

### Cervical Tissues

Tissues obtained from routine hysterectomy through the National Disease Research Interchange (Philadelphia, PA) were cultured and infected as previously described with slight modifications [5], [9]. Briefly, mucosal epithelium and underlying stroma of both ecto- and endo-cervix were separated from muscular tissue, dissected into approximately 2-mm^3^ blocks, and infected in 1.5-mL conical tubes containing 16 tissue blocks per experimental condition and 0.5 mL of viral stock. After 2 hours of incubation at 37°C, tissue blocks were gently washed three times with phosphate-buffered saline (PBS), placed on top of a collagen sponge gel into a 12 well-plate (8 blocks/well) and cultured for 12 days, with a change of medium every third day. Thus in our cultures endo- and exo-cervical tissue blocks were mixed. To distinguish p24_gag_ desorbed from viral inoculums (background) from p24 produced *de novo,* we inhibited the latter by 5 µM Lamivudine (3TC), which was replenished at each medium change. Viral replication was evaluated by p24 release and flow cytometric analysis. We considered a virus to replicate in cervical explants if the cumulative production of p24 in media bathing infected tissues was at least 100 pg higher than the p24 production of the same tissue treated with 3TC.

### Flow Cytometric Analysis

The 16 tissue blocks from each experimental condition were pooled and digested with a collagenase IV solution titered to spare cellular markers, i.e. diluted at 1.25mg/ml for the lots used in these experiments. Digestions were carried out at 37°C for 40 minutes in presence of DNAse I at 0.2 mg/ml [5]. Cells were washed, diluted in PBS, and strained through a 70 µm mesh filter (Becton Dickinson, San Jose, CA, USA). The cells were stained with live/dead blue fixable stain for 15 minutes, washed and diluted in staining buffer (PBS, 2% normal mouse serum, 2% normal goat serum, 2% normal human serum) and stained with titered amounts of fluorescently labeled monoclonal antibodies. We used anti-CD3 eFluorNC 605 (eBioscence), anti-CD4 eFluorNC 650, CD8 eFluor 450, and CD25, CD38, CD69, CD95 and HLA-DR. The presence of HIV infected cells was determined by staining with a KC57 FITC labeled anti HIV-1 p24 antibody (Beckman Coulter, Miami, FL).

### Statistical Analyses

Analyses were conducted using JMP 9.0 (SAS Institute, Cary, NC). Data were analyzed for normality using the Shapiro-Welsh test. When 3 or more groups were compared, we performed an ANOVA with the post-hoc correction of Tukey-kramer Honestly Significant Difference. When data were not normally distributed, we performed a non-parametric multiple comparison with Dunn’s correction for joined ranks. The proportion of successful infection (>100 pg p24) in tissues infected with T/F or C/R viruses were compared using Fishers’ exact test for two group comparisons or the likelihood ratio when successful infection proportions were compared across several groups. In several cases, for the reader’s information, we present both mean ± SEM and median with IQR. However, in cases of non-normal distribution of the variable, only the medians were used for statistical analysis.

## Results

In an *ex vivo* cervical tissue system we analyzed biological properties of eight HIV-1 constructs that contained *env* sequences derived from mucosally transmitted T/F HIV-1 and three constructs that contained envelopes derived from control reference HIV-1 variant (C/R) viruses: NL-SF162.ecto, NL-YU-2.ecto, and NL-BaL.ecto. All *env* sequences were expressed in otherwise isogenic NL4-3-based backbones [4]. Also, in several experiments we used two full-length T/F viruses, CH077.t and RHPA.c [6]and the laboratory-adapted HIV-1_BaL_ isolate, which we used as the reference. Earlier, we had shown that the HIV-1_BaL_ isolate and the Env-IMC cognate NL-BaL.ecto were similar in cellular tropism and virus replication in various primary target cells ([6] and unpublished]).

Cervical tissue blocks were inoculated with virus as described earlier [5] and infection was evaluated by determining the fraction of infected T cells as well as the amount of p24 released into the culture medium. Overall we performed experiments with cervical tissues from 37 donors. Each donor tissue was infected with at least one C/R virus and at least one T/F virus. According to our optimized protocol for cervical tissue infection, for any given virus stock, 16 tissue blocks per donor per condition have to be inoculated. The amount of cervical tissue obtained from individual donor did not allow for the infection of tissue from each donor with all the used viruses while keeping the number of replicates dictated by the protocol. Therefore, to increase the statistical power we pooled data from 58 infections with T/F HIV-1 variants and compared them with pooled data from 39 infections with C/R HIV-1 variants. In some experiments, we also compared the data for one T/F HIV-1 variant, NL-1051.TD12.ecto with the data for the control HIV-1 variant NL-SF162.ecto, but replicating in donor-matched cervical tissues.

In order to distinguish *de novo* HIV-1 production from the release of virus or free p24 merely adsorbed at inoculation, we treated infected tissues with the RT inhibitor 3TC. For reliably determining that the infection was productive, based on our prior experience, we defined infection to be productive if the difference between the total amount of p24 released into the medium by inoculated tissue exceeds that of the matched 3TC- treated one by at least 100 pg. Using this criterion, there were no significant differences between the fractions of tissues that supported productive infection with C/R Env-IMC, T/F Env-IMC, and T/F full length viruses (66.67%, 41.18%, and 45.45%, respectively, likelihood ratio p = 0.39).

**Figure 1:**
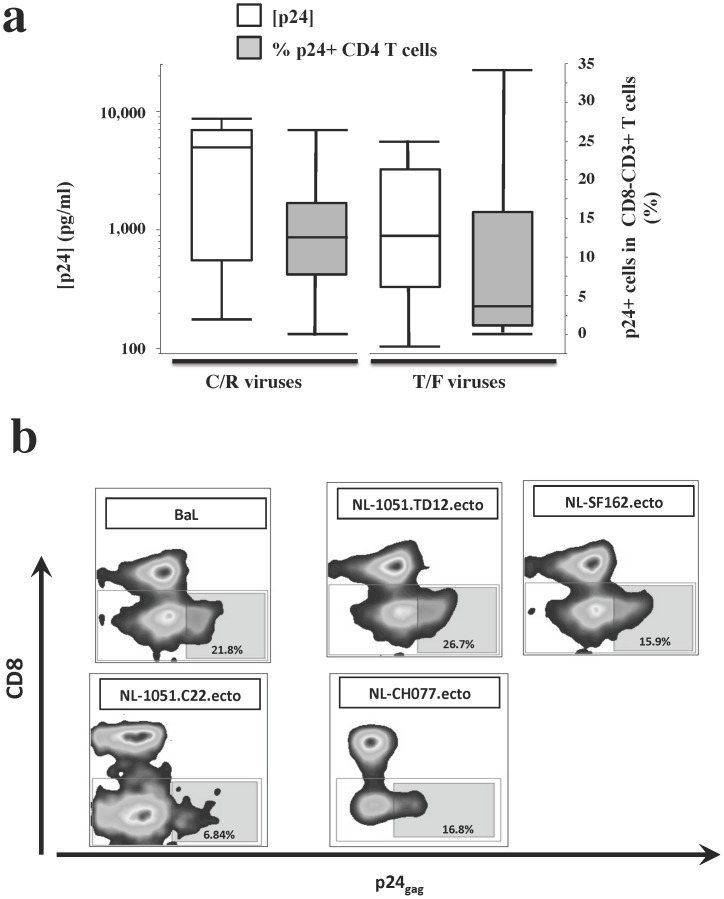
Replication of various C/R and T/F HIV-1 variants in human cervical tissue *ex vivo*. Donor-matched human cervical tissue blocks were infected *ex-vivo* with C/R and T/F viruses in presence or absence of 3TC. Culture media were collected every 3 days up to day 12 and the amount of p24 was measured. In addition, 12 days post infection, the tissues were enzymatically digested and their cells were analyzed by polychromatic flow cytometry. T cells were identified by side-scatter and staining for CD3. The CD4 T cell subset was defined as CD8^–^ T cells since the down-regulation of CD4 in HIV-1 infected cells precludes use of staining for CD4. CD4 T cell, which were infected with HIV-1 were revealed by staining for p24.(a) For each virus, cumulative values for the net p24 production ([p24] in untreated − [p24] in 3TC-treated donor-matched tissues) were computed and plotted as box plots (median, 25^th^ and 75^th^ percentiles, and range) on a log scale (left Y-axis; empty boxes; C/R virus: n = 23; T/F viruses: n = 30). The fractions of p24^+^ positive cells among CD8^−^CD3^+^ cells were plotted on a linear axis (right Y-axis; filled boxes; n = 19, and n = 14, respectively) (b). Bivariate plots showing p24 and CD8 staining in T cells; the values represent the fraction of CD8^−^CD3^+^ cells expressing p24 as defined by the plotted gate. Plots illustrate a representative experiment.

The absolute amount of the p24 released from HIV-1 infected tissue varied from donor to donor similar to what was reported previously for several other explant systems [5], [8]. The cumulative p24 tissue production ([p24] in untreated − [p24] in 3TC-treated) from C/R virus infections, was on average (mean) 3804±667pg/ml (median 4950 pg/ml, IQR [549, 6973], n = 23) whereas for T/F HIV-1 variants, the average cumulative p24 production was 2566±468pg/ml (median 892 pg/ml, IQR [325, 3350], n = 30). There were no statistically significant differences between the average cumulative amounts of p24 produced in tissues infected by either of these viruses (p = 0.058, n = 23 and n = 30 respectively) (Fig. 1).

Because of the high adsorption of some of the viruses in the tissues, in some experiments this high background may obscure the actual viral production. Therefore, for further analysis, we evaluated HIV-1 tissue infection by enumerating CD4 T cells positive for intracellular p24 by flow cytometry. At 12 or 15 days post-infection, the tissues were digested and stained for intracellular p24. We detected p24−expressing cells in tissues following exposure to all the tested HIV-1 variants (Fig. 1). To avoid the exclusion of CD4 T cells that may have down-regulated CD4 expression as a result of HIV-1 infection, we defined CD4 T cells as CD8^−^CD3^+^ cells [9].

Initially, we inoculated tissue from three donors in parallel with the T/F variant NL-1051.TD12.ecto and the C/R variant NL-SF162.ecto. We found no statistical difference between the fractions of CD4 T cells infected by these viruses (respectively 14.12±4% and 17.74±5.9%, n = 3, p = 0.74). Neither were there statistically significant differences (p = 0.08) between the fractions of p24−expressing CD4 T cells in the group of tissues infected with the C/R HIV-1 as compared to the group of tissues infected with T/F HIV-1. On average, the p24+ CD4 T cell fraction in C/R HIV-1 infected tissues constituted 12.6±1.5% (median 12.6%, IQR [7.61%–17.1%] n = 19) of total CD4 T cells, while in tissues infected with T/F viruses this fraction constituted 8.25±2.6% (median 3.76%, IQR [0.96%–15.8%], n = 14) (Fig. 1).

Next, we estimated the depletion of CD4 T cells by comparing the ratio of CD8^+^ to CD4^+^ T cells (i.e. CD8^−^CD3^+^) in infected and uninfected controls [5], [8], [10]. To pool data obtained from different donors, we normalized the data by expressing the CD4/CD8 ratio in infected tissue as a percent of the same ratio in matched uninfected controls [5], [8], [10]. Infection with C/R viruses and T/F viruses resulted in the significant depletion of tissue CD4 T cells.

First, we compared CD4 T cell depletion in donor-matched cervical tissues infected with the T/F HIV-1 NL-1051.TD12.ecto to that infected with a control C/R HIV-1 variant NL-SF162.ecto. There was no statistical difference between the CD4 T cell depletion by these viruses (respectively 27.86±28.6% and 57.07±13.8%, n = 4, p = 0.67).

**Figure 2:**
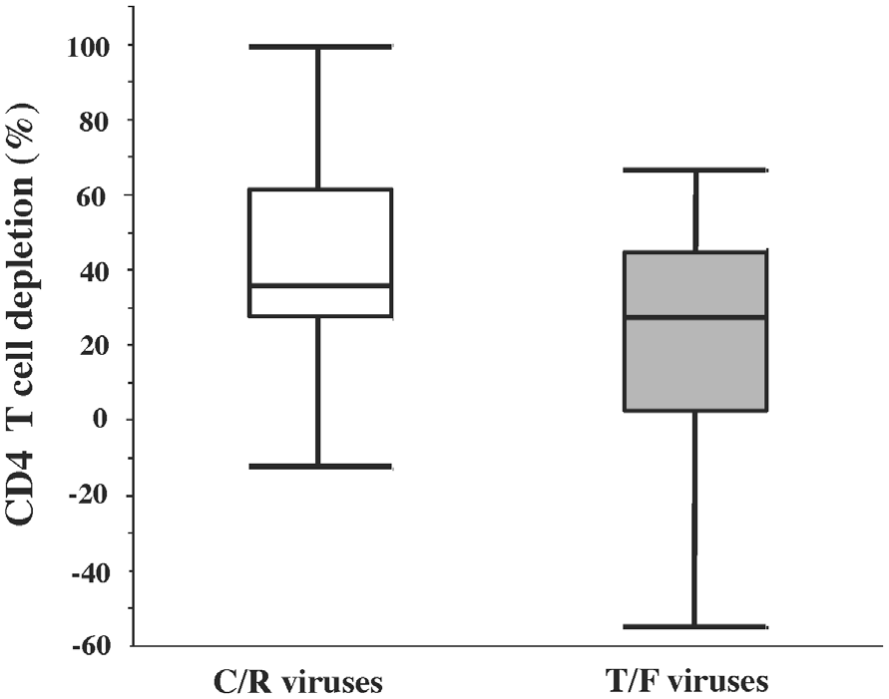
CD4 T cell depletion in C/R and T/F HIV-1 variants infected human cervical tissue *ex vivo*. Blocks of human cervical tissue were infected with C/R or T/F viruses, and cultured *ex vivo*. 12 days post infection, the tissues were enzymatically digested and their cells were analyzed by polychromatic flow cytometry. T cells were identified by staining for CD3 and side-scatter. In this subset CD4 and CD8 were identified. The depletion of CD4 T cells was estimated by comparing the ratio of CD8^+^ to CD4^+^ (CD8^−^) T cells in infected and uninfected controls, and expressing this ratio in infected tissue as a percent of the same ratio in matched uninfected control. Data showing individual experiments and summary box plots for n = 19 and n = 14 (median, 25^th^ and 75^th^ percentiles, and range of CD4/CD8 ratios in tissues infected with C/R viruses (empty boxes) and in tissues infected with T/F viruses, (filled boxes) are presented.

Next, we pooled data for all of the T/F and C/R HIV-1 variants used in the current study. These viruses respectively depleted 42.9±6.0% (median 35.26%, IQR [27.1%–61.7%], n = 19, p<0.0001) and 20.9±8.9% (median 27.32% IQR [3.01%–45.65%], n = 14, p = 0.025) of CD4 T cells. Thus, the depletion of CD4 T cells in tissues infected with these two types of HIV-1 variants was not different (p = 0.08) (Fig. 2). CD4 T cell depletion positively correlated with the proportion of infected cells in the remaining CD4 T cells as measured by flow cytometry (Spearman ρ = 0.5642, p<0.0001, n = 34). In tissues treated with 3TC, HIV-1 inoculation did not result in cell depletion: the fraction of CD4 T cells in such tissues was not statistically different from that in donor-matched uninfected tissues (n = 32, p>0.5).

**Figure 3:**
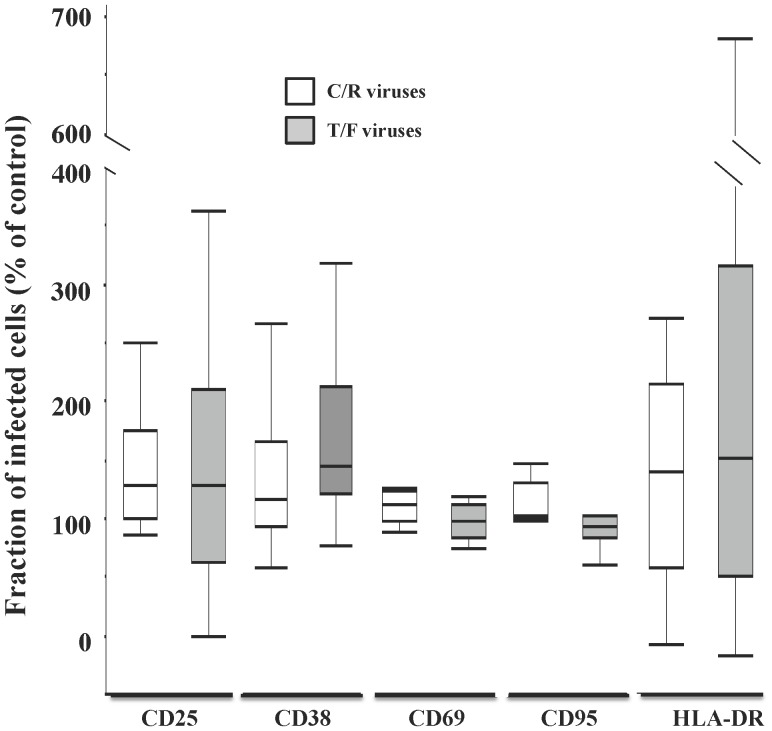
CD4 T cell activation in C/R and T/F HIV-1 variants infected human cervical tissue *ex vivo*. Blocks of human cervical tissue were infected with C/R and T/F viruses and cultured *ex vivo*. 12 days post infection, the tissues were enzymatically digested and their cells were analyzed by polychromatic flow cytometry. Cells were stained for T cell markers CD3, CD4 and CD8 as well as for the activation markers CD25, CD38, CD69, CD95, and HLA-DR. Infected cells were identified with intracellular staining for HIV-1 p24. The fraction of infected (p24^+^) CD4 T cells expressing a given activation marker was expressed as % of the fraction of T cells expressing the same marker in matched uninfected control tissue. Data showing individual experiments and summary box plots for n = 9 to 17 (median, 25^th^ and 75^th^ percentiles, and range) of activation marker expression in tissues infected with C/R viruses (empty boxes) and in tissues infected with T/F viruses (filled boxes) are presented.

Finally, we compared activation status of CD4 T cells (Fig. 3) as evaluated by the expression of the following activation markers: CD25, CD38, CD69, CD95, and HLA-DR. In uninfected tissues these markers were respectively expressed by 11.21±1.96%, 29.11±4.3%, 77.35±5.08%, 73.12±8.81%, and 7.07±1.29% of CD4 T cells (n = 24).

As with the data regarding HIV-1 infection and CD4 T cell depletion we first compared activation of T cells by their expression of CD25, CD38, and HLA-DR in donor-matched tissues infected with a T/F HIV-1 construct, NL-1051.TD12.ecto and a control C/R HIV-1 variant, NL-SF162.ecto. We found that CD25, CD38, and HLA-DR expression by p24^+^ CD4 T cells did not differ in tissues infected by these respective viruses. CD25 was expressed on respectively 20±10% and 22±9.7% (n = 3, p = 0.72) of cells infected by the HIV-1 variant NL-1051.TD12.ecto and the HIV-1 variant NL-SF162.ecto. For CD38, these fractions constituted respectively 33.4±10.7% and 40.4±10.3% (n = 3, p = 0.72), while for HLA-DR, these fractions were 6.03±2.5% and 8.75±3.8% (n = 3, p = 0.38), respectively. These results were confirmed when we analyzed the expression of activation markers in the group of tissues infected with T/F HIV-1 variants as compared to the group infected with C/R HIV-1 variants. In tissues infected with C/R HIV-1 variants, CD25, CD38, CD69, CD95, and HLA-DR were respectively expressed by 15.03±2.67%, 24.27±4.25%, 78.17±2.77%, 80.15±9.14%, and 7.61±1.58% of the p24^+^ CD4 T cells. In tissues infected with T/F viruses, these markers were expressed by 17.44±3.57%, 28.39±5.26%, 75.04±4.83%, 80.16±12.12%, and 5.8±1.58% of p24^+^ CD4 T cells.

In order to distinguish the effects of viral infection from the normal variation of marker expression between donor tissues, for each matched tissue, we calculated the level of expression in infected (p24^+^) CD4 T cells as the percent of the level of expression in the matched non–infected tissue. This analysis revealed that, in tissues infected with C/R viruses, 140±11.7% (median 127.23%, IQR [100.8%, 174.4%], n = 17, p = 0.004) of HIV-1–infected CD4 T cells expressed CD25 compared to those in control uninfected tissues. Similarly, larger fractions of HIV infected T cells expressed the activation markers CD38, CD95 and HLA-DR: respectively 153±31.2% (n = 17, p = 0.0253), 123±14.2% (n = 9, p = 0.012) and 203±33.72% (n = 17, p = 0.003) relative to these fractions in donor matched control tissues. In contrast, there was no difference between CD69-expression in HIV-1 infected CD4 T cells as compared to cells in uninfected control tissues (n = 9, p = 0.055). In tissues infected with T/F viruses, our analysis revealed that the fraction of HIV-infected CD4 T cells was enriched in cells expressing CD38 and HLA-DR (p = 0.007), but not CD25, CD69, or CD95 (p>0.28). HIV-1–infected T cells expressing CD38 and HLA-DR constituted, respectively 161±20.9% (median 144.23%, IQR [121.8%, 211.5%], n = 11, p = 0.0068) and 277.79±85.17% (median 191.21%, IQR [95.5%, 348.57%], n = 11, p = 0.0244) of the number CD4 T cells expressing these markers in control tissues. In tissues inoculated either with T/F or C/R HIV-1 variants and treated with 3TC, there was no increase in the fractions of CD4 T cells expressing activation markers compared to donor-matched control tissues (p = 0.074, p = 0.91). Infection by both C/R and T/F HIV-1 variants resulted in activation of not only productively infected (p24^+^) but also of uninfected (p24^−^) bystander CD4 T cells, as shown by the higher expression of some of the tested markers by the latter cells compared to their expression by CD4 T cells in uninfected tissues. This difference reached statistical significance for CD25. However, this activation of uninfected bystander CD4 T cells was not different in tissues infected by T/F or C/R HIV-1 variants (p>0.77 ).

Thus, in tissues infected either with C/R or T/F HIV-1 variants, the fraction of activated CD4 T cells, either infected or bystander, was larger than the fraction of activated CD4 T cells in matched uninfected tissues. In general more CD4 T cells infected with C/R or T/F viruses are activated than CD4 T cells in matched uninfected tissues. However, there was no statistical difference between the numbers of CD4 T cells infected with C/R and T/F HIV-1 expressing any of the studied activation markers (p>0.09).

## Discussion

It was observed more than a decade ago that not all HIV-1 variants are capable of establishing new infection upon transmission: while both CCR5- tropic (R5) and CXCR4-tropic (X4) HIV-1 variants are present in various body fluids, only R5 viruses are apparently transmitted and dominate the early stages of HIV-1 disease [11]. Such precision suggests the existence of an effective barriers (“gatekeepers”) that selects R5 over X4 HIV-1 transmission [12].

Recently, the effectiveness of the gatekeeping mechanisms that select transmitted viruses among R5 HIV-1 variants has been highlighted: Application of single genome amplification in samples collected shortly after HIV infection, unequivocally demonstrated that, only one or at most few HIV particles initiate the infection in the course of sexual transmission [13]. However, which viral properties are essential for transmission remains unclear. We recently reported on the generation of 10 Clade B T/F infectious molecular clones [6] and showed that these T/F HIV-1 variants, including some used here, are macrophage-tropic but display generally low but measurable monocyte-derived macrophage replicative capacity, as defined by [14]. While these T/F viruses share this phenotype with other “low” macrophage-tropic R5 variants like HIV-1 JRCSF, they differ from widely used reference viruses, including BaL, YU-2 and SF162, which are prototypic macrophage-tropic with very high macrophage replicative capacity. This finding highlights that in order to gain a better understanding of mechanisms involved in mucosal transmission, it is important to test the most relevant virus strains in the most physiological model systems. Here, we attempted to investigate biological properties of T/F HIV-1 variants by studying their transmission to cervical tissue *ex vivo*. This *ex vivo* system is closer to the situation *in vivo* than single-cell cultures, as the integral structure of cervical tissue and presumably many aspects of cell-cell interactions are preserved.

In this study we focused predominantly on the envelopes of T/F HIV-1 that were expressed in a pNL4-3 backbone. Specifically, we expressed full-length *env* genes in this backbone in which the heterologous *env* sequence (either T/F or control) comprised gp120 and the external portion of gp41. In several experiments we also used full-length T/F HIV-1 (molecular clones). We infected tissues by inoculating them with equal volumes of viral stocks, whereby the TCID_50_ varied between different stocks by approximately four-fold, although this variation did not correlate with whether a stock was from a T/F or C/R HIV-1 variant. However, the TCID_50_ evaluated in one system (TZM-bl) cannot be directly translated into another system, but serves merely as a measure of reference. Moreover, a virus stock with a higher TCID_50_ evaluated in a cell line may replicate less efficiently in tissues than a virus stock with a lower TCID_50_ [7]. Nevertheless, measuring TCID_50_ in cervical tissue is impractical due to the limitations in the amount of tissue obtainable from one donor and to the variability between the donors. Furthermore, we have observed that the normal variability in replication of a given virus in tissues from different donors is higher than four-fold. To avoid a possible bias we pooled data on replication of T/F HIV-1 variants in numerous donor tissues. We also pooled data for the C/R HIV-1 variants. In addition, we chose two individual constructs, one T/F (NL-1051.TD12.ecto), one C/R (NL-SF162.ecto), isogenic except for their *env* sequences, to compare in detail their infection in three donor-matched cervical tissues. While availability of sufficient numbers and amounts of donor tissue limits the scope of our study, we nevertheless believe such studies to be relevant and informative.

Ideally, the biological properties of the T/F envelopes that have been transmitted from a male donor to a recipient should be compared with the non-transmitted envelopes of HIV-1 variants present in the semen of the same donor at the time of transmission. However, the cohorts from which the studied infectious molecular clones of T/F variants have been derived do not include sexual partners (donors) of the recipients of the T/F HIV-1. Therefore, we studied the biological properties of the HIV-1 variants with T/F Env alongside with the molecular clones with HIV-1 envelopes of widely utilized control reference viruses, as well as the cognate isolates themselves, taken from different biological compartments: HIV-1_BaL_ (isolated from lung), and HIV-1_SF162_ (isolated from cerebrospinal fluid). We argued that the use of these C/R laboratory-adapted HIV-1 variants as controls should maximize the chance of detecting unique phenotypic characteristics of T/F viruses. Nevertheless, our study did not reveal striking differences between C/R and T/F HIV-1 envelopes in infection of cervical tissue that pointed towards a T/F phenotype related gatekeeping mechanism(s).

Although the rate of HIV-1 transmission *ex vivo* is much higher than that *in vivo,* not every cervical tissue inoculated with HIV-1 supported productive infection. Why some cervical tissues were not infected by HIV-1 remains to be studied and may be related to the stage of menstrual cycle at which they were isolated [G. Poli, personal communication] and/or the expression of innate restriction factors. Whichever the gatekeeping mechanisms that protect the tissues from infection are, the rates of transmission of C/R and T/F HIV-1 variants were not different in our model system.

Using p24 release into the culture medium as a read-out, some tissues may support replication of HIV-1 at a level that we did not consider reliably indicative of *de novo* virus production since the p24 amount measured may merely represent a slow release of virions adsorbed during inoculation. To exclude these tissues from further analysis, we established a formal criterion: tissue was considered to support productive HIV-1 infection if the amount of the released virus exceeded the amount of the released adsorbed virus by 100 pg. Although this criterion is somewhat arbitrary, in our experience the total amount of cumulative production of virus over 12 days of culture should be not lower than this amount. To determine the cumulative *de novo* production, we blocked HIV-1 infection with the NRTI 3TC and measured the amount of virus released. The amount of 3TC we applied seems to block HIV-1 infection, as neither CD4 T cell depletion nor CD4 T cell activation were observed in these tissues.

In tissues that were productively infected we evaluated the efficiency of this infection by measuring the release of p24 in the culture medium and by enumerating p24^+^ CD4 T cells with flow cytometry. By both these criteria there were no statistically significant differences between tissues inoculated with C/R and T/F HIV-1 variants.

T cell depletion is a hallmark of HIV-1 infection. All HIV-1 variants employed here significantly deplete cervical tissue of CD4 T cells, and with similar efficiency. As expected the magnitude of T cell depletion is proportional to the efficiency of infection, in our case to the number of infected cells in the tissue. Neither when we compared CD4 T cell depletion in NL-SF162.ecto– and NL-1051.TD12.ecto–infected donor matched tissues, nor when we compared all T/F and C/R HIV-1 variants as groups, were there statistically significant differences.

It is known that activated CD4 T cells preferentially support productive HIV-1 infection and that HIV-1 infection may activate bystander cells [15]. This was confirmed in this study: there were more activated cells (as evaluated by the expression of various activation markers) among HIV-1 infected T cells than in controls. Both T/F and C/R HIV-1 variants replicated predominantly in these activated cells. And again, neither when we compared CD4 T cell activation in NL-SF162 ecto– and NL-1051.TD12.ecto–infected donor matched tissues, nor when we compared all T/F and C/R HIV-1 variants, was there a general difference in CD4 T cell activation.

Thus, the biological properties of T/F and C/R HIV-1 variants as revealed in their infection of cervical tissues *ex vivo* were similar. Obviously, it is possible that the subtle differences between the T/F and C/R HIV-1 variants are not revealed in *ex vivo* tissues, which, although closer to the *in vivo* situation than isolated cell cultures may fail to reflect important systemic factors such as recruitment of new cells to the site of infection, cell trafficking to the draining lymph nodes, etc. Moreover, unlike *in vivo*, the tissue is not polarized and thus the inner cells are not protected by the epithelial layer, although according to some studies HIV-1 is transmitted directly to cell targets in the inner layers through lesions in the epithelium [16]. If this is the case, our tissue model faithfully represents the *in vivo* situation.

In this study we focused on the infection of cervical T cells, which have also been reported to be the earliest detectable infected cells in human genital mucosa ex-vivo [17]. However, according to some reports dendritic cells (DCs) and macrophages also may play an important role in the early events of HIV infection. Unlike intestinal macrophages, genital mucosal macrophages are permissive to HIV-1 productive infection [18] and are thought to play a role in the early events of HIV transmission [19]. In the vagina, the initial infection is established in the outer epithelium where intraepithelial T cells bind and take up HIV-1 independently of Langerhans cells [20]. The latter, while they remain non-productively infected, can mediate the infection of T cells [21]. Simillarly, DCs that have captured HIV-1 through their sugar binding receptors [22] can transfer the virus through viral synapses [23], to remote CD4 T cells [24]–[26]. Nevertheless, a direct evidence for the implication of mucosal dendritic cells in the transmission of HIV-1 in vivo is still lacking. Moreover, in the studies of SIV transmission to non-human primates it was shown that the predominant primary HIV-1 targets are T lymphocytes [1], [27]. These cells express high levels of CD4 and CCR5 and are depleted in the vagina of infected animals [28].

While our data did not reveal differences in the infection patterns of T cells between T/F and C/R HIV-1 variants, it is conceivable that some minor subpopulations of tissue cells, may be differentially infected by these two groups of viruses but produce too a small quantity of p24 to affect the bulk production of T cells and thus to be noticed. Nevertheless, taking into account all the above reservations regarding our experimental system, our data did not reveal any particular features of T/F HIV-1 variants that made them strikingly different from the reference HIV-1 variants used in the current experiments.

For ultimate determination of whether there are HIV-1 quasispecies that preferentially overcome HIV gatekeeper mechanisms or whether HIV-1 particles are transmitted stochastically, further studies utilizing pairs of transmitted and non-transmitted HIV-1 variants from the same host are necessary. Results from a recent transmission pair study [29] indicate argued that HIV-1 transmission is not solely stochastic, but there are sequences that seem to be transmitted preferentially. In contrast, in a recent *in vitro* study [30] no differences were found between T/F and C/R HIV-1 in their transmission efficiency across cervical tissue or in the genetic diversity of these viruses before and after transmission. More studies are needed to understand the biological propertied of HIV-1 variants that transmit infection.
